# HDAC6 Inhibition Alleviates CLL-Induced T-Cell Dysfunction and Enhances Immune Checkpoint Blockade Efficacy in the Eμ-TCL1 Model

**DOI:** 10.3389/fimmu.2020.590072

**Published:** 2020-11-23

**Authors:** Kamira Maharaj, John J. Powers, Melanie Mediavilla-Varela, Alex Achille, Wael Gamal, Steven Quayle, Simon S. Jones, Eva Sahakian, Javier Pinilla-Ibarz

**Affiliations:** ^1^ Department of Immunology, H. Lee Moffitt Cancer Center & Research Institute, Tampa, FL, United States; ^2^ Cancer Biology PhD Program, University of South Florida & H. Lee Moffitt Cancer Center & Research Institute, Tampa, FL, United States; ^3^ Department of Molecular Medicine, Morsani College of Medicine, University of South Florida, Tampa, FL, United States; ^4^ Cue Biopharma, Cambridge, MA, United States; ^5^ Regenacy Pharmaceuticals, Inc., Waltham, MA, United States; ^6^ Department of Malignant Hematology, H. Lee Moffitt Cancer Center & Research Institute, Tampa, FL, United States

**Keywords:** chronic lymphocytic leukemia, immune checkpoint, PD-1, PD-L1, HDAC6, histone deacetylase, B cell, T cell

## Abstract

Development of chronic lymphocytic leukemia (CLL) is associated with severe immune dysfunction. T-cell exhaustion, immune checkpoint upregulation, and increase of regulatory T cells contribute to an immunosuppressive tumor microenvironment. As a result, CLL patients are severely susceptible to infectious complications that increase morbidity and mortality. CLL B-cell survival is highly dependent upon interaction with the supportive tumor microenvironment. It has been postulated that the reversal of T-cell dysfunction in CLL may be beneficial to reduce tumor burden. Previous studies have also highlighted roles for histone deacetylase 6 (HDAC6) in regulation of immune cell phenotype and function. Here, we report for the first time that HDAC6 inhibition exerts beneficial immunomodulatory effects on CLL B cells and alleviates CLL-induced immunosuppression of CLL T cells. In the Eμ-TCL1 adoptive transfer murine model, genetic silencing or inhibition of HDAC6 reduced surface expression of programmed death-ligand 1 (PD-L1) on CLL B cells and lowered interleukin-10 (IL-10) levels. This occurred concurrently with a bolstered T-cell phenotype, demonstrated by alteration of coinhibitory molecules and activation status. Analysis of mice with similar tumor burden indicated that the majority of T-cell changes elicited by silencing or inhibition of HDAC6 *in vivo* are likely secondary to decrease of tumor burden and immunomodulation of CLL B cells. The data reported here suggest that CLL B cell phenotype may be altered by HDAC6-mediated hyperacetylation of the chaperone heat shock protein 90 (HSP90) and subsequent inhibition of the Janus kinase (JAK)/signal transducer and activator of transcription (STAT) pathway. Based on the beneficial immunomodulatory activity of HDAC6 inhibition, we rationalized that HDAC6 inhibitors could enhance immune checkpoint blockade in CLL. Conclusively, combination treatment with ACY738 augmented the antitumor efficacy of anti-PD-1 and anti-PD-L1 monoclonal antibodies in the Eμ-TCL1 adoptive transfer murine model. These combinatorial antitumor effects coincided with an increased cytotoxic CD8^+^ T-cell phenotype. Taken together, these data highlight a role for HDAC inhibitors in combination with immunotherapy and provides the rationale to investigate HDAC6 inhibition together with immune checkpoint blockade for treatment of CLL patients.

## Introduction

Chronic lymphocytic leukemia (CLL) is the most common leukemia in the western world, usually affecting older adults (1). Currently, it remains incurable unless bone marrow transplant is feasible. CLL is characterized by accumulation of leukemic CD5^+^ B cells in lymph nodes, blood, and bone marrow, resulting in severe immune dysfunction. Although recent clinical developments in B-cell receptor (BCR) inhibitors, B-cell lymphoma 2 (BCL-2) inhibitors, and immunotherapies have revolutionized treatment strategies, patients may be intolerant, become resistant, or ultimately fail therapy (2, 3). CLL cells depend on crosstalk with a unique, immunosuppressive microenvironment that ultimately contributes to progression and plays a role in response to therapy. In the microenvironment, dysfunctional CLL T cells support malignant B cells by providing extrinsic survival signals (4). T-cell exhaustion and upregulation of immune checkpoints permit tumor immune evasion (5). Increased regulatory T cells (Tregs) suppress effector responses. Based on these observations, it has been postulated that reversal of T-cell dysfunction may restore T-cell mediated antitumor activity. Detailed reviews of CLL T-cell dysfunction can be found here (6, 7).

Several studies have explored the idea of reversing T-cell exhaustion in CLL. Brusa et al. initially identified that the PD-1/PD-L1 axis drives T-cell exhaustion in CLL (8). This study reported that PD-L1^hi^ CLL cells and associated closely with PD-1^+^ T cells in lymphoid tissue. Gassner et al. found that pretreatment of Eμ-TCL1 splenocytes with recombinant PD-1 blocking fragments reduced CLL engraftment when these cells were adoptively transferred to wildtype recipients (9). This study concluded that antitumor effects were due to T-cell mediated tumor cell lysis. In another study, McClanahan et al. characterized CLL-specific PD-1 induction on Eμ-TCL1 T cells. Interestingly, PD-1^+^ T cells exhibited variable effector function (10). This group also reported antitumor efficacy of PD-L1 blocking antibody in the Eμ-TCL1 model and suggested that combination with immunomodulatory agents should be explored (11).

Anti-PD-1 antibodies pembrolizumab and nivolumab have demonstrated clinical activity in solid tumors and are currently in trial for B-cell malignancies. Although CLL cells express immune checkpoints, anti-PD-1 antibodies have not been efficacious in all patients, and the reason is suspected to be a complicated, chronically suppressed immune microenvironment. In a Phase II trial, pembrolizumab elicited responses in 4 out of 9 CLL patients who had undergone Richter transformation (RT), but 0 out of 16 patients who had relapsed after prior therapy with ibrutinib without RT (12). Increased PD-L1 on B cells and a trend of increased PD-1 on T cells were detected in responders versus non-responders before therapy. However, expression of other checkpoints before and after therapy was not determined. Nivolumab, in combination with ibrutinib, also elicited responses in CLL patients that underwent RT (13). These results suggest that PD-1 blockade may be useful for RT patients. For the non-RT, relapsed CLL patients, further optimization will be required. Anti-PD-L1 antibody atezolizumab is currently undergoing clinical trial for CLL patients in combination with anti-CD20 antibody obinutuzumab, and BCL-2 inhibitor venetoclax (14); however, final results are not yet reported. We speculated that in relapsed CLL patients, multiple mechanisms of B-cell driven immune suppression might overwhelm responses to immune checkpoint therapy. We, therefore, hypothesized that novel combination approaches that target immune suppression could maximize efficacy of immune checkpoint blockade.

Roles for histone deacetylases (HDACs) in immunobiology of cancer have been reported by our group and others. Although HDACs were initially described to epigenetically modulate histone proteins, it is now well documented that they also interact with and modulate the function of non-histone proteins. Histone deacetylase 6 (HDAC6) inhibition has been found to augment immunogenicity of melanoma cells through inhibition of STAT3-mediated PD-L1 transcription (15). HDAC6 was also found to regulate the activity of the STAT3/IL-10 pathway in professional antigen-presenting cells (APCs), leading to increased antigen presentation (16). More recently, we have described upregulation of HDAC6 protein levels in CLL patients’ B cells and direct antitumor activity of selective HDAC6 inhibition in murine CLL (17). Whole exome sequencing analysis showed that silencing of HDAC6 in Eμ-TCL1 B cells elicited changes in immune-related signaling networks, including antigen presentation and cytokine signaling (17). In the current study, we therefore sought to investigate the immunomodulatory role of HDAC6 in CLL and test the rational combination of a selective HDAC6 inhibitor with PD-1/PD-L1 blockade in the Eμ-TCL1 adoptive transfer model of murine CLL.

## Materials and Methods

### Ethics

The studies involving laboratory animals were reviewed and approved by the Institutional

Animal Care and Use Committee, Research Integrity and Compliance, University of South Florida, Tampa, FL. CLL patient samples: All participants gave written Institutional Review Board-approved informed consent for blood to be used for research. Blood was collected at the H. Lee Moffitt Cancer Center and Research Institute. CLL patients were diagnosed according to the guidelines of the International Workshop on Chronic Lymphocytic Leukemia (1). Patient characteristics are detailed in **Table 1**

**Table 1:** CLL patient characteristics.

	Age	Gender	Cytogenetics	CD38 Status	ZAP70 Status	IGVH Status
CLL 1	51	M	11q del	Negative	Positive	Unmutated
CLL 2	63	M	13q del	Negative	Positive	Unmutated
CLL 3	73	F	Trisomy 12	Negative	Negative	Unmutated
CLL 4	67	M	13q del	Positive	Positive	Unmutated
CLL 5	71	M	Trisomy 12	Positive	Negative	Mutated
CLL 6	46	F	13q del	Unknown	Negative	Mutated
CLL 7	75	F	Trisomy 12	Positive	Negative	Mutated
CLL 8	74	M	13q del	Negative	Negative	Mutated

del, deletion; CD38, cluster of differentiation 38; ZAP70, zeta-chain associated protein kinase; IGVH, immunoglobulin variable heavy chain.

### Cell Culture

Cells were cultured in RPMI supplemented with 10% fetal bovine serum, 5% penicillin-streptomycin, 5% non-essential amino acids and 1% Mycozap, incubated at 37°C with 5% CO_2_. OSU-CLL was authenticated before use. Cell isolations were performed using magnetic separation kits (StemCell Technologies, Vancouver, CA).

### Reverse Transcriptase Quantitative Polymerase Chain Reaction

Total RNA was isolated using Trizol reagent (Invitrogen, Carlsbad, CA). Complementary DNA was synthesized from RNA using iScript Reverse Transcriptase (BioRad, Hercules, CA). Primers against human *T-bet*, *Eomes*, *IL-2* (Qiagen, Venlo, Netherlands) were used together with iScript Reaction Mix (BioRad, Hercules, CA).

### Cytotoxicity Assay

CD8^+^ effector cells and CD19^+^ target cells were isolated from splenocytes by magnetic separation *via* negative selection using anti-mouse isolation kits (StemCell Technologies, Vancouver, CA). Ligand-loaded CD19^+^ target cells were cocultured together with effectors in various ratios for 4 h. Supernatant was removed from culture and europium reagent was added to detect released ligand. Cytotoxicity was measured by the DELFIA time-resolved fluorescence cell cytotoxicity assay according to manufacturer’s instructions (PerkinElmer, Waltham, MA).

### Antigen Presentation Assay

Eμ-TCL1 B cells from 6-month old transgenic leukemic mice were isolated from splenocytes by magnetic separation and pre-treated with ACY738 for 24 h at concentrations that elicited less than 35% cell death, leaving a majority of viable cells (determined by trpan-blue exclusion) in accordance with previously published data (17). Eμ-TCL1 B cells were then centrifuged and washed in PBS to remove drug and non-viable cell content. An equal number of viable Eμ-TCL1 B cells from each dose condition were then loaded with ovalbumin (OVA) peptide, and co-cultured with isolated transgenic OTII CD4^+^ T cells in a 1:2 B cell to T cell ratio at a final density of 3 × 10^6^ cells per ml. IFNγ secretion into supernatant was quantified by cytokine bead array analysis (BD CBA Mouse Th1/Th2, BD Biosciences, San Jose, CA).

### CLL Mouse Model

An HDAC6-deficient CLL murine model (Eμ-TCL1/HDAC6KO) was generated by crossing HDAC6KO (18) and Eμ-TCL1 (19) (C57BL/6 background) mice. Eμ-TCL1 mice are referred to as euTCL1 or euTCL1/HDAC6KO in figures. All Eμ-TCL1 and Eμ-TCL1/HDAC6KO mice were homozygous for T-cell leukemia 1 (*TCL1*) gene, and all mice harboring HDAC6KO were homozygous for the knockout as confirmed by genotyping. For the accelerated CLL model, several aged leukemic Eμ-TCL1 mice (aged leukemic was defined as >9 months of age and showing >70% CD5^+^ B cells of total viable lymphocytes by flow analysis) were euthanized, and their splenocytes were pooled. Freshly obtained splenocytes were then resuspended in phosphate-buffered saline and adoptively transferred *via* tail vein into 6- to 8-week-old C57BL/6 wildtype (WT) mice at 25 × 10^6^ splenocytes per mouse. CLL induction was confirmed at 3 weeks after adoptive transfer by high complete blood count and a significantly greater CD19^+^ B220^+^ CD5^+^ B lymphocyte population in peripheral blood than in peripheral blood from a healthy age-matched WT cohort. Groups were randomized before treatment. For survival analyses, mice were monitored until death or euthanasia resulting from disease symptoms such as lethargy, difficulty moving, lack of grooming, and enlarged spleen and/or lymph nodes. Mice were kept in pathogen-free conditions and handled in accordance with Guidelines for Animal Experiments requirements.

### Flow Cytometry

For murine CLL immunophenotyping, 100 ul of peripheral blood was freshly obtained from submandibular bleeds. Spleen tissue was also freshly obtained from mice sacrificed for immunophenotyping analysis. Spleens were homogenized into a single-cell suspension, washed in PBS and resuspended in FACS buffer for staining. Red blood cells were lysed prior to staining with ACK lysis buffer (Lonza, Walkersville, MD) according to manufacturer’s protocol. Cells were stained with surface antibodies for 1 h at room temperature. AccuCheck Counting Beads (Life Technologies, Frederick, MD) were utilized to obtain cell counts according to manufacturer’s protocol. For phosphorylated proteins, cells were stimulated then fixed in 1% paraformaldehyde (BD Phosflow Fix Buffer I) and permeabilized in ice-cold 90% methanol/PBS prior to staining with phospho-specific antibody or isotype control for 1 h at 4°C. For cytoplasmic proteins, cells were stimulated with PMA/ionomycin in the presence of GolgiStop for 5 h, fixed/permeabilized with BD Cytofix/Cytoperm kit prior to staining for 1 h at room temperature. Cytokines were determined with BD CBA Mouse Th1/Th2, BD Biosciences, San Jose, CA and protocol was conducted according to manufacturer’s instructions. Assays were run on LSRII (BD Biosciences, Franklin Lakes, NJ) or iQue Screener (Intellicyt, Albuquerque, NM) flow cytometers and analyzed with FlowJo software version 10.6.1 (Becton, Dickinson and Company, Ashland, OR). tSNE and xshift plugins were utilized within FlowJo software. A list of antibodies used can be found in **Supplemental Table 1**.

### Immunoblotting

Samples were analyzed by sodium dodecyl sulfate polyacrylamide gel electrophoresis (SDS-PAGE), followed by transfer to nitrocellulose membrane, blocking with 5% nonfat milk and incubation with the indicated antibodies overnight at 4°C. Blots were developed using LI-COR system. Cell lysis was performed with radioimmunoprecipitation assay buffer (10 mM Tris-HCl, pH 7.4, 150 mM NaCl, 1% NP-40, 0.5% sodium deoxycholate, 0.1% SDS, and 10% glycerol) supplemented with protease inhibitors (Roche, Basel, Switzerland). Protein quantities were determined using the Qbit 4 fluorometer and the Invitrogen Qbit Protein Assay Kit according to manufacturer’s instructions (Thermofisher Scientific, Waltham, MA). A list of antibodies used can be found in **Supplemental Table 1**.

### Inhibitors and Blocking Antibodies

ACY738 (20) and ACY1215 (15) was supplied by Acetylon Pharmaceuticals. Anti-PD-1 and anti-PD-L1 monoclonal antibodies were commercially obtained (BioXcell, Lebanon, NH). For *in vitro* assays, inhibitors were dissolved in sterile dimethylsulfoxide (DMSO) and stored at −20°C until used. ACY738 was administered orally in chow at 25mpk (mg/kg) per day. Anti-PD-1 and anti-PD-L1 antibodies were diluted in sterile phosphate buffered saline and administered by intraperitoneal injection at 3 mpk, 3 times per week, for a total of 2 weeks.

### Statistical Analysis

Statistical significance between data sets was determined by unpaired, two-tailed, or Student’s *t* test if data were normally distributed, or using a Mann-Whitney U unpaired test if the data were not normally distributed. For groups of 3 or more, one-way ANOVA followed by Tukey’s multiple comparisons test was used if the data were normally distributed, or a Kruskal-Wallis test was used if the data were not normally distributed. Two-way ANOVA followed by Tukey’s multiple comparisons test was used for groups with more than 1 time point. Kaplan-Meier survival curves were compared using the log-rank test. Overall survival was defined as time from adoptive transfer of leukemic cells until death or euthanasia. A *p* value <.05 was considered significant. All analyses were conducted using GraphPad Prism software version 8 or Microsoft Excel version 16.

## Results

### HDAC6 Silencing or Inhibition Disrupts Regulatory CLL B Cell Characteristics

**Figure 1:**
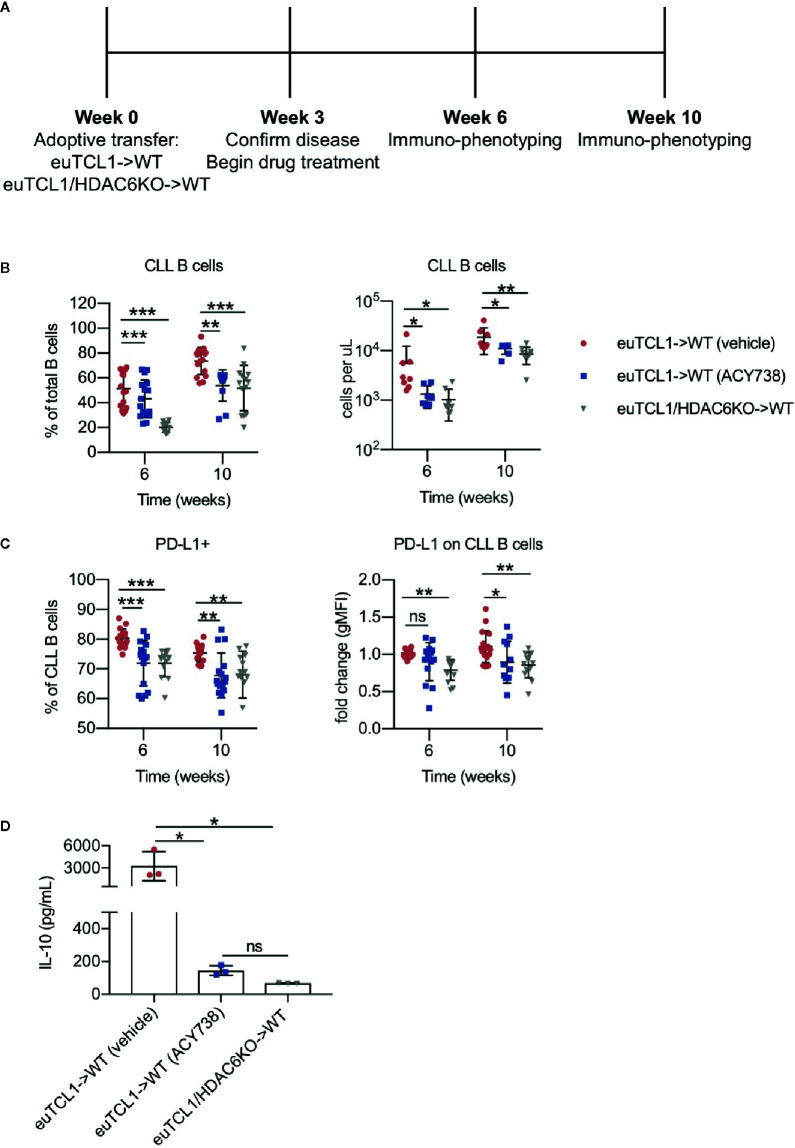
Immunomodulatory effects of HDAC6 on CLL B cells **(A)** Timeline showing experimental protocol. **(B)** Flow cytometry to detect tumor burden in fresh murine PBMCs. CLL B cells were gated as CD3^-^ CD19^+^ B220^lo^ IgM^hi^ CD5^+^ cells. Total B cells were gated as CD3^-^ CD19^+^ B220^+^ cells. **(C)** Percentages of PD-L1^+^ CLL B cells and expression of PD-L1 on CLL B cells. gMFI: geometric mean fluorescence intensity. Percentages are compiled from 2 independent experiments, n = 12–14 mice per group. Counts are shown from 1 representative experiment, n = 8 mice per group. **(D)** Plasma was collected from submandibular bleeds and IL-10 was quantified by cytokine bead array assay, n = 3 mice per group. Graphs display mean + SD. *p < 0.05, **p < 0.005, ***p < 0.005. ns, not significant.

CLL B cells exhibit a regulatory phenotype that suppresses immune cell responses in the tumor microenvironment, similar to the normal regulatory B cell (Breg) subset (21). Both CLL B cells and normal Bregs suppress effector T-cell function through interleukin (IL)-10 secretion and expression of immune checkpoint PD-L1. The Eμ-TCL1 transgenic murine model spontaneously develops CLL over its lifetime due to expression of the *TCL1* gene, specifically in B cells (22). This model recapitulates immune dysfunction typical of CLL patients and has revealed insights into development of the CLL microenvironment. Eμ-TCL1 B cells exhibit Breg characteristics and retain immunoregulatory function when adoptively transferred (23). To examine the immunomodulatory role of HDAC6 in CLL, we utilized the Eμ-TCL1 adoptive transfer murine CLL model. Aged-matched Eμ-TCL1 or Eμ-TCL1/HDAC6KO splenocytes from transgenic leukemic mice were adoptively transferred into syngeneic immunocompetent wildtype recipients. After CLL engraftment was established, mice were randomized, and Eμ-TCL1 recipients were treated orally with a selective HDAC6 inhibitor, ACY738 (20), or vehicle (**Figure 1A**). Malignant CLL cells were gated as previously reported (17) according to surface expression of CD19, B220, IgM, and CD5 in peripheral blood mononuclear cells (PBMCs). Both ACY738-treated mice and Eμ-TCL1/HDAC6KO recipient mice (HDAC6KO) demonstrated delayed CLL progression over time compared to vehicle-treated mice (**Figure 1B**), in agreement with our previous report (17). PD-L1 expression was found to be downregulated on malignant B cells of ACY738 and HDAC6KO groups compared to the vehicle group at 6 and 10 weeks post-adoptive transfer (**Figure 1C**). Plasma levels of IL-10 were also found to be lower in ACY738 and HDAC6KO groups compared to the vehicle group (**Figure 1D**). Additionally, a separate cohort of CLL-bearing mice treated as in **Figure 1A** were sacrificed at Week 7. Tumor burden analysis in fresh splenocytes confirmed reduced tumor burden and reduced percentages of PD-L1^+^ CLL cells of ACY738-treated and HDAC6KO groups (**Figures S2A, B**). To assess whether PD-L1 downregulation was independent of tumor burden, mice with similar tumor burden from the 10-week time point in **Figure 1B** were compared. Percentages of PD-L1^+^ CLL cells were also significantly downregulated in ACY738-treated and HDAC6KO mice of similar tumor burden compared to vehicle controls (**Figure S3C**).

### HDAC6 Silencing or Inhibition Alleviates the Dysfunctional CLL T-Cell Phenotype

**Figure 2:**
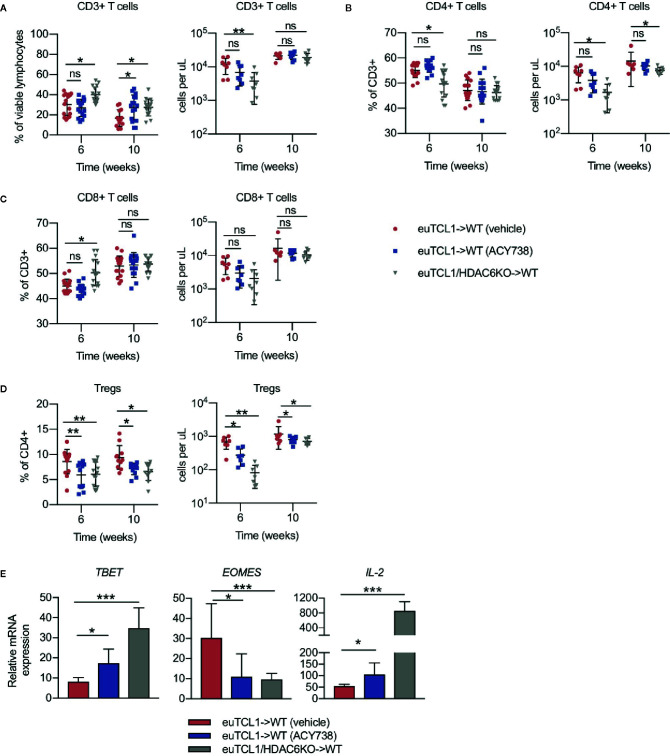
Effects of HDAC6 deficiency on CLL T-cell dysfunction. Flow cytometry to detect immune populations in fresh PBMCs of mice in **Figure 1**. **(A–C)** Percentages and counts of CD3^+^, CD4^+^ and CD8^+^ T cells. **(D)** Percentages and counts of Tregs out of CD4^+^ gate. Tregs were gated as CD4^+^ CD25^HI^ CD127^LO^. Percentages are compiled from 2 independent experiments, n = 12–14 mice per group. Counts are shown from 1 representative experiment, n = 8 mice per group. **(E)** Relative mRNA expression (ddCT) of *T-bet*, *Eomes*, and *IL-2* in isolated splenic T cells of mice from indicated groups, n = 3 mice per group. Normalized to 18s control gene. Graphs display mean + SD. *p < 0.05, **p < 0.005, ***p < 0.0005. ns, not significant.

Previous studies demonstrated longitudinal development of CLL-induced T-cell subset changes in the adoptive transfer Eμ-TCL1 model compared to aged-matched wildtypes (10). Documented changes included skewing of CD4/CD8 ratio, naïve/antigen-experienced ratio, T helper (Th)1/2 ratio, increased exhaustion, and decreased effector function. However, some studies suggest that CLL T cells can be reprogrammed by therapeutic intervention to exert antitumor function (24, 25). Here, we sought to examine the T-cell compartment of adoptive transfer mice described in **Figure 1**. T cells were gated according to the strategy depicted in **Figure S1**. Relative to the vehicle group, increases in CD3^+^ T-cells and alterations to CD4/CD8 ratio were observed in HDAC6KO and ACY738-treated groups (**Figures 2A–C**). Treg percentages and absolute counts were also decreased in ACY738 and HDAC6KO groups compared to vehicle group (**Figure 2D**). Similar T-cell changes were noted in splenocytes of mice sacrified at Week 7 (**Figures S2C–F**). Next, Th1/2 subsets were evaluated. q-RT-PCR was performed to assess expression of Th lineage-specific factors in isolated splenic CD4^+^ T cells. Compared to vehicle, ACY738 and HDAC6KO groups favored the Th1 phenotype, showing increased expression of Th1 differentiation factor *T-bet*, as well as Th1 cytokine *IL-2*. Expression of Th2 differentiation factor *Eomes* was decreased on T cells from the HDAC6KO and ACY738 groups (**Figure 2E**) compared to vehicle. These changes to the T-cell compartments were overall indicative of a lower tumor burden in these mice.

**Figure 3:**
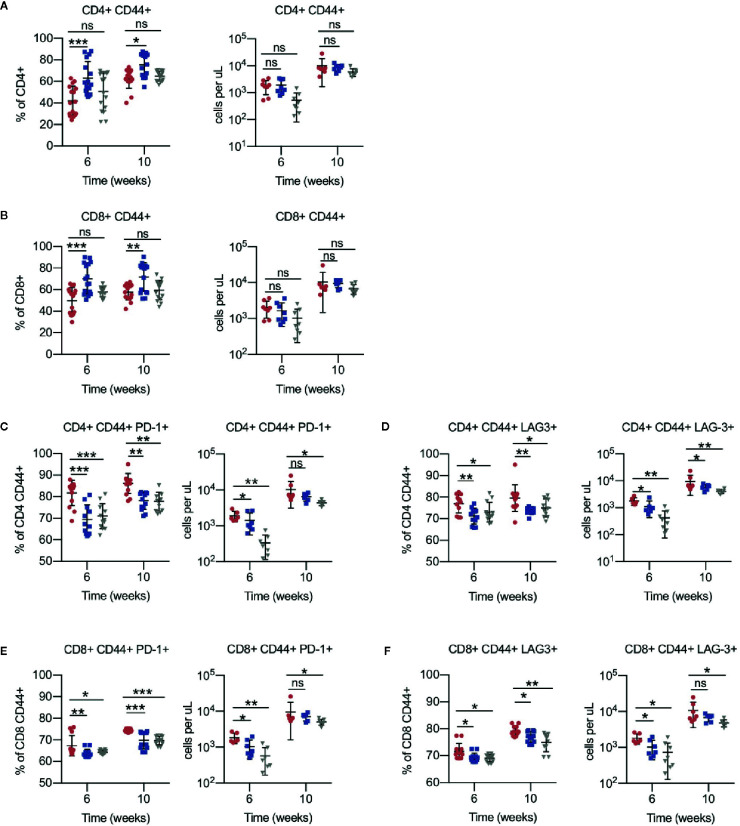
Effects of HDAC6 deficiency on antigen-experienced CLL T-cell compartment. **(A)** Percentages and counts of antigen-experienced CD44^+^ out of CD4^+^ gate. **(B)** Percentages and counts of antigen-experienced CD44^+^ out of CD8^+^ gate. **(C, D)** Percentages and counts of PD-1^+^ and LAG3^+^ cells out of CD44^+^ CD4^+^ gate. **(E, F)** Percentages and counts of PD-1^+^ and LAG3^+^ cells out of CD44^+^ CD8^+^ gate. Percentages are compiled from 2 independent experiments, n = 12–14 mice per group. Counts are shown from 1 representative experiment, n = 8 mice per group. Graphs display mean + SD. *p < 0.05, **p < 0.005, ***p < 0.0005. ns, not significant.

To assess antigen-experienced and exhausted T cells, CD44^+^ PD-1^+^ and LAG-3^+^ cells were gated. Compared to vehicle, the antigen-experienced CD44^+^ fraction was consistently increased by approximately 1.5-fold in CD4^+^ and CD8^+^ T cells of ACY738-treated mice (**Figures 3A, B**), but not HDAC6KO recipients. This difference may be evidence of an ongoing antitumor T-cell response in the ACY738 group. PD-1^+^ and LAG-3^+^ percentages and cell counts were significantly decreased in both CD44^+^ CD4^+^ and CD44^+^ CD8^+^ T cells of ACY738 and HDAC6KO groups (**Figures 3C–F**), indicating a less exhausted phenotype in the antigen-experienced fraction. Represenative flow plots are shown in **Figures S3A, B**. Similar trends were noted in splenocytes of mice sacrificed at Week 7 (**Figures S2G–J**). To assess whether the CD44^+^ T-cell changes were independent of tumor burden, mice with similar tumor burden from the 10-week time point represented in **Figure 1B** were compared. Percentages of CD4^+^ CD44^+^ and CD8^+^ CD44^+^ T cells were increased ACY738-treated mice of similar burden compared to vehicle group (**Figure S3D**). CD44^+^ LAG-3^+^ T cells, but not CD44^+^ PD-1^+^ T cells, were decreased in ACY738-treated group compared to vehicle group (**Figures S3E, F**).

### HDAC6 Inhibition in CLL Cells Promotes T-Cell Engagement

**Figure 4:**
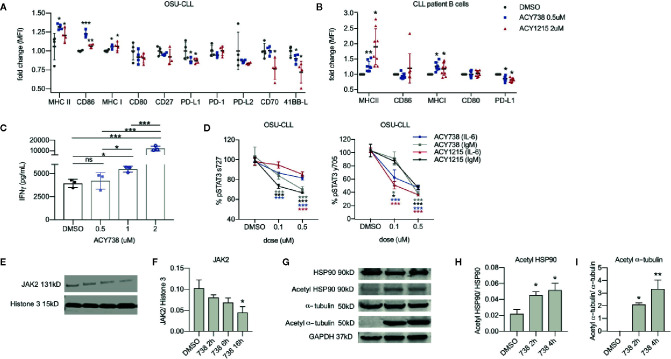
Effects of HDAC6 inhibition on T-cell engagement and JAK/STAT signaling in CLL **(A)** Surface expression of antigen presentation and co-stimulatory/co-inhibitory markers in OSU-CLL cell line incubated with selective HDAC6 inhibitors or DMSO for 24 h. Data is compiled from 4 independent experiments. All values are expressed as fold change normalized to the mean of DMSO controls. Graph displays mean + SD. **(B)** Surface expression of antigen presentation and co-stimulatory/co-inhibitory markers on the surface of CLL patient B cells (n = 8 patients) incubated with selective HDAC6 inhibitors or DMSO for 24 h. CLL cells were denoted by expression of CD19, CD20 and CD5. All values are expressed as fold change normalized to DMSO controls. Each patient sample was normalized to its own DMSO control to account for inter-patient variability in antigen expression. Graph displays mean + SD. **(C)** Antigen presentation assay. Eμ-TCL1 B cells were pre-treated with ACY738 at indicated concentrations for 24 h. They were then washed and an equal number of viable cells from each dose condition were loaded with OVA peptide and co-cultured with OTII CD4^+^ T cells for 24 h in a 1:2 B cell:T cell ratio. IFNγ secretion into supernatant was quantified by cytokine bead array. Representative of 3 independent experiments. Graph displays mean + SD. **(D)** Phospho-STAT3 protein levels in OSU-CLL treated with ACY738 for 24 h were detected by flow cytometry. Cells were plated at a density of 1 × 10^6^ per ml and stimulated with soluble anti-human IL-6 at 20 ng/ml for 15 min or plate-bound anti-human IgM at 10 ug/ml for 50 min. All values are expressed as a percentage of DMSO controls. Data are compiled from 3 independent experiments. Graphs display mean + SD. **(E)** Immunoblotting analysis for JAK2 protein in OSU-CLL cells treated with ACY738 at 0.5uM collected at indicated time points. **(F)** Densitometry quantifying JAK2 expression, normalized to histone 3 loading control. **(G)** Immunoblotting analysis for acetylation of HSP90 and α-tubulin in OSU-CLL cells treated with ACY738 at 0.5uM collected at indicated time points. **(H, I)** Densitometry quantifying acetylation of HSP90 and α-tubulin. Normalized to total protein control. Immunoblots shown are representative of three independent experiments and densitometry graphs display data compiled from 3 independent experiments. Graphs display mean + SD. *p < 0.05, **p < 0.005, ***p < 0.0005. ns, not significant.

To further investigate the direct effects of HDAC6 inhibition on CLL B cell engagement with T cells, the patient-derived cell line, OSU-CLL, was utilized for *in vitro* assays. We examined the expression of functional surface proteins involved in T-cell engagement, including antigen presentation, B-cell activation, co-stimulatory, and coinhibitory markers (26). In OSU-CLL cells treated with ACY738, expression of MHCI, MHCII, and CD86 were increased, while expression of PD-L1 and 41BB-L were consistently decreased compared to DMSO-treated cells (**Figure 4A** and **Figure S4A**). Our results were confirmed with ACY1215 (ricolinostat), a less potent, but clinically available selective HDAC6 inhibitor (15). Additionally, CLL patient B cells treated with ACY738 and ACY1215 were analyzed for expression of surface markers. Expression of MHCII and MHCI were notably increased in ACY-treated cells, while PD-L1 expression was decreased (**Figure 4B** and **Figure S4B**). CLL patient characteristics are shown in **Table 1**. The observed differences support a less inhibitory B-cell phenotype with increased antigen presentation capability. To confirm this hypothesis, we conducted an antigen presentation assay adapted from Cheng et al. (16). Aged leukemic Eμ-TCL1 B cells were isolated from splenocytes and pre-treated with ACY738. An equal number of viable Eμ-TCL1 B cells from each dose condition were then loaded with OVA peptide, and co-cultured with transgenic OTII CD4^+^ T cells, which only recognized OVA peptide presented through MHCII by the leukemic B cells. T-cell activation was then quantified by interferon gamma (IFNγ) secretion. Eμ-TCL1 B cells pre-treated with ACY738 dose-dependently elicited greater T-cell activation compared to DMSO control (**Figure 4C**). These results support the notion that HDAC6 inhibition can improve antigen presentation by CLL B cells, thereby eliciting increased T-cell activation.

### HDAC6 Inhibition Alters JAK/STAT Signaling in CLL Cells

We then investigated the role of HDAC6 in molecular pathways sustaining the Breg phenotype. Studies by other groups suggest mechanistic involvement of STAT3 signaling in CLL B cell immunoregulatory function (27). These studies report that elevated IL-6 and IL-10 cytokine levels provoke JAK/STAT signaling in CLL B cells, activating STAT-driven transcription of *PD-L1* and *IL-10*. JAK/STAT signaling is also activated downstream of BCR signaling in CLL cells (28), and ibrutinib, a BTK/ITK inhibitor, has been shown to downregulate STAT3 phosphorylation in CLL patient B cells (29). Considering this data, we stimulated OSU-CLL cells with IL-6 or IgM to mimic intrinsic and extrinsic survival signals, inducing STAT3 phosphorylation at serine 727 and tyrosine 705. Following previously established protocols, we performed intracellular flow cytometry analysis to detect phosphorylation of STAT3 (30). Selective HDAC6 inhibition decreased STAT3 phosphorylation at both residues compared to DMSO-treated controls in OSU-CLL cells (**Figure 4D**). Prior literature has reported that acetylation of chaperone heat shock protein 90 (HSP90) drives degradation of JAK2 protein through endoplasmic reticulum (ER) stress pathway (31), and that HDAC6 deacetylates HSP90 (32) in other cell types. We then hypothesized that HDAC6 inhibition induces HSP90 hyperacetylation and provokes JAK2 degradation, preventing STAT3 activity in CLL cells. In agreement with this hypothesis, JAK2 protein was decreased over time in OSU-CLL cells treated with ACY738 (**Figures 4E, F**), while acetylation of HSP90 at lysine 294 was increased (**Figures 4G, H**). This occurred concurrently with hyperacetylation of α-tubulin, a known cytoplasmic target of HDAC6 (**Figures 4H, 4I**).

### Combination of HDAC6 Inhibition and PD-1/PD-L1 Blockade Augments Antitumor Efficacy and T-Cell Cytotoxicity

**Figure 5:**
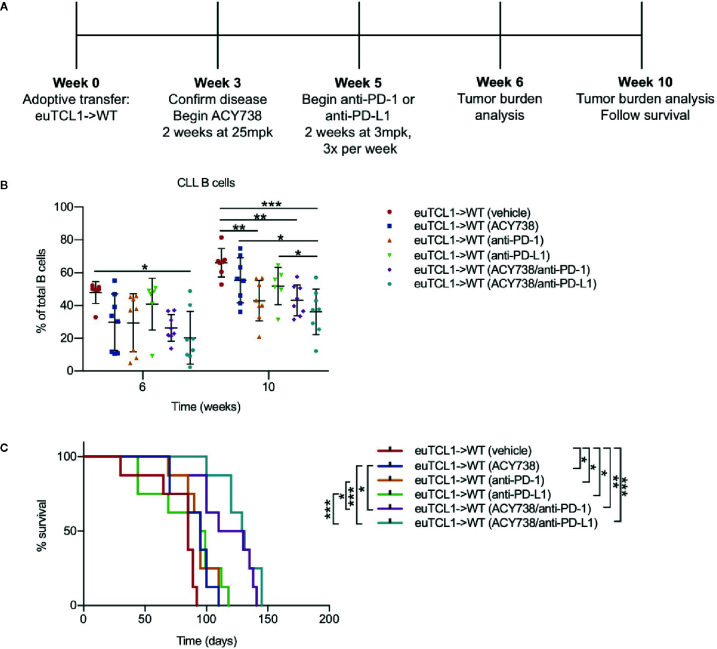
Combinatorial antitumor efficacy of ACY738 and PD-1/PD-L1 blockade in CLL. **(A)** Timeline showing experimental protocol. **(B)** Flow cytometry to detect tumor burden in fresh PBMCs. CLL B cells were gated as CD3^-^ CD19^+^ B220^lo^ IgM^hi^ CD5^+^ cells. Total B cells were gated as CD3^-^ CD19^+^ B220^+^ cells. **(C)** Kaplan-Meier curve showing survival analyses for mice in panel **(A)**. n = 8 mice per group. Representative of three independent experiments. Graphs display mean + SD. *p < 0.05, **p < 0.005, ***p < 0.0005.

Given the results showing that HDAC6 inhibition relieved CLL T-cell dysfunction *in vivo*, we rationalized that it may be therapeutically beneficial to combine HDAC6 inhibition with immune checkpoint blockade in CLL in order to augment T-cell mediated antitumor activity. To test this hypothesis, Eμ-TCL1 adoptive transfer mice were treated first with ACY738 or vehicle for 2 weeks, followed by anti-PD-1 or anti-PD-L1 for 2 weeks. The experimental protocol is summarized in **Figure 5A**. Both ACY738/anti-PD-1 and ACY738/anti-PD-L1 combination treatments significantly delayed CLL progression compared to vehicle. Tumor burden in ACY738/anti-PD-L1 group was decreased compared to anti-PD-L1 group (**Figure 5B**) in peripheral blood, and both ACY738/anti-PD-1 and ACY738/anti-PD-L1 groups exhibited decreased tumor burden compared to anti-PD-1 or anti-PD-L1 groups, respectively, in spleen tissue (**Figure S5A**), suggesting that these combinations elicited a beneficial antitumor effect. Notably, each single agent treatment demonstrated survival advantage compared to vehicle group, and the combination of ACY738/anti-PD-1 or ACY738/anti-PD-L1 increased survival probability more than anti-PD-1 or anti-PD-L1 alone (**Figure 5C**).

**Figure 6:**
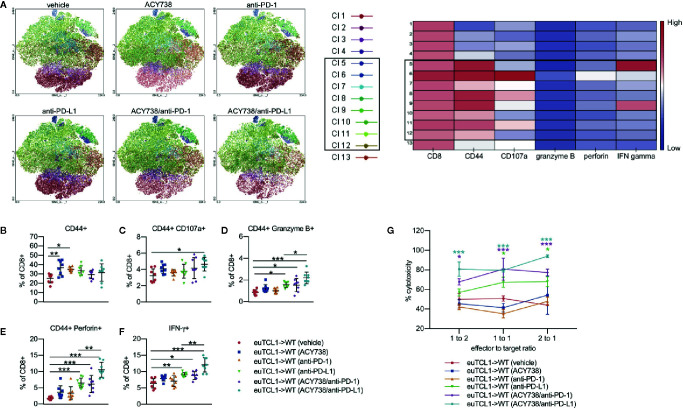
Combination of ACY738 and PD-1/PD-L1 blockade enhances cytotoxic CLL T-cell response. **(A–F)** Mice treated as in Figure 4 were sacrificed at week 7 post-adoptive transfer and splenocytes were stimulated with PMA/ionomycin in the presence of GolgiStop for 4 h. Cytotoxic cellular proteins were detected by flow cytometry. **(A)** Unbiased clustering analysis by tSNE. Xshift analysis was performed to determine clusters of cells based on differential expression of markers. **(B)** Antigen-experienced CD44^+^ fraction out of CD8^+^ T cells. **(C–F)** Percent positive for indicated cytotoxic markers out of antigen-experienced CD8^+^ CD44^+^ fraction. n = 6–8 mice per group. **(G)** Europium-release cytotoxicity assay. CD8^+^ effector T cells were incubated with CD19^+^ ligand-loaded target B cells for 4 h. Fluorescence was quantified to detect ligand released into supernatant by dead target cells. % lysis is expressed normalized to positive control wells containing target cells treated with lysis buffer, and background fluorescence was subtracted using negative control wells containing viable, ligand-loaded target cells only. n = 4 mice per group. Comparisons indicated in **(G)** are versus vehicle group. Graphs display mean + SD. *p < 0.05, **p < 0.005, ***p < 0.0005. Cl, cluster.

Immune checkpoint blockade *via* anti-PD-1 treatment has been shown to elicit antitumor activity in a CLL model by increasing tumor-specific cytolysis (9). To investigate the effects of combination treatments on cytotoxic T-cell activity, groups of mice were sacrificed upon completion of treatment, and stimulated splenocytes were assessed by flow cytometry for functional markers of cytotoxicity. Unbiased clustering analysis by t-stochastic neighbor embedding (tSNE) and xshift algorithms showed several clusters with high expression of CD44 (**Figure 6A**). These CD44^HI^ cells also contained the majority of cells with high expression of CD107a (clusters 6, 8, 11), granzyme B (cluster 6), perforin (cluster 6), and IFN-γ (clusters 5 and 9). Relative expression of each marker in each identified cell cluster is visualized in the heatmap corresponding to the xshift analysis (**Figure 6A**). We therefore inferred that these clusters may represent cells with cytotoxic potential. Quantification of these populations based on xshift analysis demonstrated that some clusters with cytotoxic potential (6, 9, 10, 11, 12) appeared enriched in treatment groups (**Figure S5B**). The expression distribution of each marker in the tSNE plot is shown in **Figure S6**.

Traditional gating of antigen-experienced CD44^+^ CD8^+^ cells, the fraction containing tumor-specific T cells, was then performed. Compared to vehicle, percentages of granzyme B^+^, perforin^+^ and IFNγ^+^ cells were increased in mice treated with single agent anti-PD-L1, but not anti-PD-1 (**Figures 6B–F**). Combination of ACY738 with anti-PD-1 or anti-PD-L1 further increased expression of these markers compared to anti-PD-1 or anti-PD-L1 alone, suggesting that pretreatment with ACY738 augmented the ability of T-cells to mount a cytotoxic response. *p* values for multiple comparisons of each marker among treatment groups can be found in **Table 2**. Finally, we performed a functional cytotoxicity assay utilizing splenocytes from all treatment groups. Isolated effector CD8^+^ Eμ-TCL1 T cells were cocultured with autologous target CD19^+^ Eμ-TCL1 B cells. Results showed that tumor cell lysis increased significantly in anti-PD-L1 and both combination treatment groups compared to vehicle group (**Figure 6G**). Altogether, these analyses support the notion that combination of ACY738 with PD-1/PD-L1 blockade potentiated T-cell cytotoxicity in murine CLL, leading to greater antitumor effects and survival benefit.

**Table 2:** Multiple comparisons analysis for T cells expressing cytotoxic markers depicted in **Figures 6B–F**.

	CD44	CD107a	Granzyme B	Perforin	IFNγ
euTCL1->WT (vehicle) vs. euTCL1->WT (ACY738)	0.007	0.337	0.485	0.371	0.279
euTCL1->WT (vehicle) vs. euTCL1->WT (anti-PD-1)	0.030	0.953	0.975	0.576	0.932
euTCL1->WT (vehicle) vs. euTCL1->WT (anti-PD-L1)	0.095	0.782	0.011	0.0002	0.006
euTCL1->WT (vehicle) vs. euTCL1->WT (ACY738/anti-PD-1)	0.767	0.263	0.022	0.001	0.019
euTCL1->WT (vehicle) vs. euTCL1->WT (ACY738/anti-PD-L1)	0.335	0.021	<0.0001	<0.0001	<0.0001
euTCL1->WT (ACY738) vs. euTCL1->WT (anti-PD-1)	0.993	0.846	0.901	0.999	0.829
euTCL1->WT (ACY738) vs. euTCL1->WT (anti-PD-L1)	0.901	0.976	0.496	0.061	0.586
euTCL1->WT (ACY738) vs. euTCL1->WT (ACY738/anti-PD-1)	0.173	>0.9999	0.647	0.120	0.832
euTCL1->WT (ACY738) vs. euTCL1->WT (ACY738/anti-PD-L1)	0.537	0.788	0.0002	<0.0001	<0.0001
euTCL1->WT (anti-PD-1) vs. euTCL1->WT (anti-PD-L1)	0.996	0.998	0.076	0.027	0.071
euTCL1->WT (anti-PD-1) vs. euTCL1->WT (ACY738/anti-PD-1)	0.436	0.769	0.127	0.057	0.174
euTCL1->WT (anti-PD-1) vs. euTCL1->WT (ACY738/anti-PD-L1)	0.857	0.157	<0.0001	<0.0001	<0.0001
euTCL1->WT (anti-PD-L1) vs. euTCL1->WT (ACY738/anti-PD-1)	0.739	0.947	1.000	1.000	0.998
euTCL1->WT (anti-PD-L1) vs. euTCL1->WT (ACY738/anti-PD-L1)	0.985	0.344	0.035	0.002	0.002
euTCL1->WT (ACY738/anti-PD-1) vs. euTCL1->WT (ACY738/anti-PD-L1)	0.979	0.862	0.019	0.001	0.001

Color scale represents *p* value.

## Discussion

CLL B cells employ multiple tumor immune evasion strategies, including loss of antigen presenting function, upregulation of coinhibitory surface ligands, and secretion of suppressive cytokines. Several reports suggest bi-directional crosstalk between CLL B cells and immune cells in the microenvironment, resulting in a unique immune landscape not only permitting immune evasion, but actively supporting malignant cell transformation, propagation and survival (4). The mechanisms controlling the immunoregulatory functions of CLL B cells and their targetable vulnerabilities are yet to be fully understood, and this is an ongoing area of research. Previous literature has established roles for HDAC family members in control of the immune response (33), and the ability of HDACs to regulate the immune system suggest potential immunotherapeutic avenues. Previous studies by our group and others have demonstrated increased expression of HDAC6 in CLL B cells (17, 34). In the current study, we demonstrated an immunomodulatory role for HDAC6 in CLL B cells. Genetic silencing of HDAC6 in Eμ-TCL1 B cells delayed disease progression, downregulated PD-L1 expression, and reduced plasma IL-10 plasma levels in recipient mice when compared to controls. Similar results were seen in mice administered with the HDAC6 selective inhibitor, ACY738. Further analysis demonstrated that HDAC6 inhibition in CLL cells promoted antigen presentation and T-cell engagement.

Prior literature has described constitutive phosphorylation of STAT3 in CLL cells, and there is a known role of JAK/STAT in regulating PD-L1 and IL-10 expression (28). In an interesting recent report, Kondo et al. characterized the ability of ibrutinib to suppress CLL Breg function through STAT3-mediated inhibition of the PD-1/PD-L1 pathway. In other cell types, it has been demonstrated that HSP90 acetylation facilitates ER stress-mediated JAK2 degradation (31), and HSP90 is a known target of HDAC6 (32). In light of these studies, we focused on whether HDAC6 inhibition could affect JAK/STAT signaling in CLL cells. Indeed, selective HDAC6 inhibition lead to hyperacetylation of HSP90 in CLL cells, alongside reduction of JAK2 and phospho-STAT3. This highlights one possible mechanism to explain the immunomodulatory effects of HDAC6 on the CLL Breg phenotype. This study does not rule out other possible mechanisms, such as direct interaction of HDAC6 with STAT3 or localization of HDAC6 to the PD-L1 promoter which has been presented in other studies (16, 35).

Accumulation of dysfunctional T cells alongside CLL development has been well documented, but the specific roles of CLL T-cell subsets as supporters or drivers of disease are still debated (7). Recent studies offer evidence that dysfunctional CD8^+^ T-cell phenotype is driven by a CLL-specific response, leading to chronic antigen exposure and activation-induced exhaustion (5, 36–38). These studies note expression of multiple inhibitory receptors on CD8^+^ T cells in CLL, and suggest that strategies to restore CD8^+^ T-cell function could have therapeutic value. Many reports describe the ability of targeted therapies to beneficially reshape the T-cell microenvironment. Long et al. reported that ibrutinib treatment markedly increased total T cell numbers in CLL patients and enriched effector/effector memory proportions (39). These investigators also noted reduced percentages of PD-1 and CTLA-4-expressing T cells in CLL patients treated with ibrutinib or acalabrutinib. Weerdt et al. reported that after 1 year of venetoclax-based therapy, frequencies of tumor-supportive T-follicular helper cells, Tregs and PD-1^+^ CD8^+^ T cells were significantly decreased in CLL patients (40). Other studies have demonstrated that inhibition or silencing of PI3Kδ in preclinical CLL models modulates T-cell subsets, particularly Tregs (41–43).

**Figure 7:**
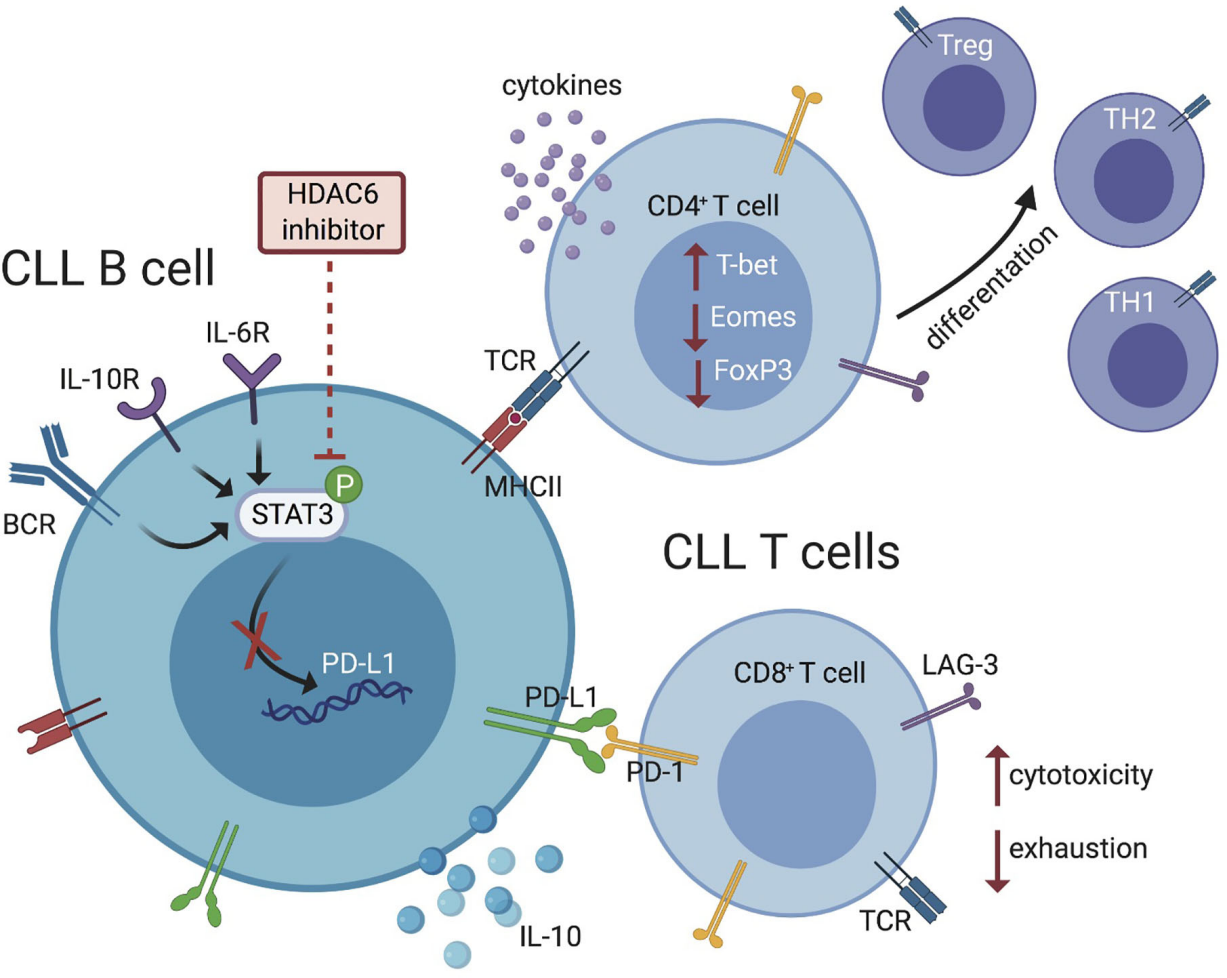
Schematic representation summarizing the effects of HDAC6 inhibition on immunosuppressive mechanisms within the CLL microenvironment. HDAC6 inhibition disrupts regulatory CLL B-cell phenotype and function *via* JAK/STAT signaling, allowing T-cell activation and antitumor responses.

Prior studies in solid tumors show that selective HDAC6 inhibition can influence tumor cell immunogenicity and beneficially alter T-cell phenotypes, reducing suppressive populations and enhancing effector function (35, 44). These studies also demonstrate that HDAC6 inhibition can induce expansion of memory subsets in tumor-infiltrating T lymphocytes (35, 44, 45). In our current work, T-cell subset analyses indicated a less exhausted, Th1 driven, effector T-cell phenotype in Eμ-TCL1/HDAC6KO mice and ACY738-treated Eμ-TCL1 mice. Despite changes to immunoregulatory B-cell phenotype, HDAC6 silencing or inhibition did not induce tumor-free survival in CLL-bearing mice, but rather delayed tumor progression. Since CLL cells depend on multiple survival and immune evasion strategies, we speculate that CLL cells in this model may eventually develop resistance mechanisms that allow disease progression, such as (1) clonal evolution of CLL B cells to depend on survival signaling unaffected by HDAC6 (2) mutation in BCR signaling kinases or (3) compensatory mechanisms through HDAC family members or epigenetic modulators with overlapping function. Analysis of mice with similar tumor burden indicated that the majority of T-cell changes elicited by silencing or inhibition of HDAC6 *in vivo* are likely secondary to decrease of tumor burden and immunomodulation of CLL B cells. Taken together, these data support a model where CLL cells in the HDAC6i-treated and Eμ-TCL1/HDAC6KO groups exerted less immunoregulatory pressure on the microenvironment, preventing typical CLL-induced T-cell dysfunction and allowing tumor immune surveillance to some extent (visually summarized in **Figure 7**).

The contribution of the PD-1/PD-L1 axis to CLL progression has been thoroughly documented in preclinical and clinical studies (8). Although anti-PD-1 treatment has been shown to elicit beneficial responses in solid tumors and some B-cell lymphomas, this is not yet the case for CLL patients (46). Novel methods to target multiple mechanisms of immune suppression to stimulate T-cell responses and studying the role of the microenvironment in response to immunotherapy will be valuable to improve clinical benefit in the future. Several groups are exploring this concept in preclinical models. Wierz et al. utilized adoptively transferred Eμ-TCL1 mice to identify and characterize immune cell composition from the tumor microenvironment by mass cytometry (47). These authors found that dual PD-1/LAG-3 blockade in Eμ-TCL1 mice effectively reduced tumor load and restored an antitumor immune response. Hanna et al. found that ibrutinib in combination with PD-1/PD-L1 blockade in Eμ-TCL1 mice reduced tumor burden and increased percentage of cytotoxic CD8^+^T cells over ibrutinib single agent treatment (48). In our current work, we found that HDAC6 inhibition downregulated, but did not fully ablate PD-L1 expression on CLL B cells, and simultaneously enhanced MHC-restricted antigen presentation. We therefore tested the rational combination of HDAC6 inhibition and PD-1/PD-L1 axis blockade in the adoptive transfer Eμ-TCL1 model. Results demonstrated that pretreatment with ACY738 in combination with anti-PD-1 or anti-PD-L1 elicited better antitumor responses and survival benefit. T cells derived from vehicle-treated mice demonstrated a cytolytic response *ex vivo*, which was not improved by ACY738 or anti-PD-1 alone. Conversely, anti-PD-L1 treatment alone induced a more potent cytolytic T-cell response, which was further augmented in both sets of combination-treated mice. This study therefore highlights an integral role for HDAC inhibitors in combination with immunotherapeutic agents and provides rationale to test selective HDAC6 inhibitors in combination with immune checkpoint blocking antibodies in CLL patients.

## Data Availability Statement

All datasets presented in this study are included in the article/**Supplementary Material**.

## Ethics Statement

All animal studies were reviewed and approved by Internal IACUC.

## Author Contributions

KM designed and conducted experiments, performed data analysis, interpreted results, and wrote the manuscript. JP designed and conducted experiments and performed data analysis. MM-V, AA, WG conducted experiments. SQ and SJ served as advisors. ES and JP-I conceived the project, designed experiments, interpreted results and revised the manuscript. All authors contributed to the article and approved the submitted version.

## Funding

This work was supported by a Leukemia and Lymphoma Society Translational Research program grant (TRP-2283-14 Grant ID 6476-15) and Acetylon Pharmaceuticals, and in part by the Flow Cytometry Core Facility at Moffitt Cancer Center, an NCI-designated Comprehensive Cancer Center (P30-CA076292). The funder bodies were not involved in the study design, collection, analysis, interpretation of data, the writing of this article or the decision to submit it for publication.

## Conflict of Interest

Author JP-I received research funding from the company Acetylon Pharmacueticals. SQ was employed by the company Acetylon Pharmaceuticals and Cue Biopharma. SJ was employed by the company Acetylon Pharmaceuticals. SJ is employed by the company Regenacy Pharmaceuticals.

The remaining authors declare that the research was conducted in the absence of any commercial or financial relationships that could be construed as a potential conflict of interest.
