# Supplementary material for: HDAC6 Inhibition Alleviates CLL-Induced T-Cell Dysfunction and Enhances Immune Checkpoint Blockade Efficacy in the Eμ-TCL1 Model
Source: Front Immunol. 2020 Nov 23;11:590072. doi: 10.3389/fimmu.2020.590072 (PMC7719839; doi:10.3389/fimmu.2020.590072)
Supplement: Supplementary file 1 [file DataSheet_1.pdf]

Supplementary Material

1.1 Supplementary Figures

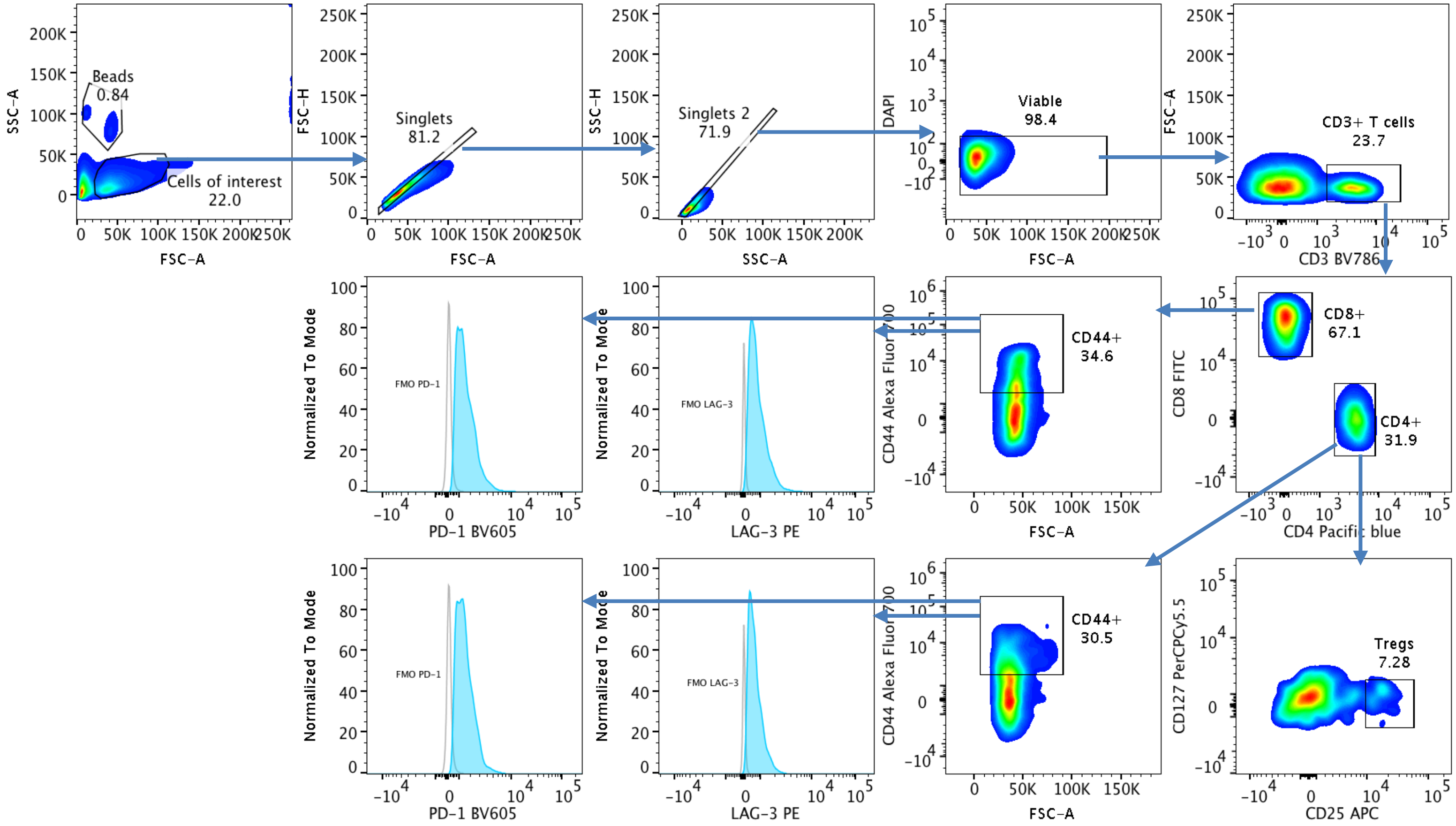

Supplementary Figure 1. Gating strategy to identify murine CLL T-cell subsets in immunophenotyping analysis. Singlet viable CD3^{+} cells were gated on CD4^{+} and CD8^{+}. CD4^{+} cells were then gated on CD25^{HI} CD127^{LO} cells to identify Tregs. CD44^{+} cells were gated to identify antigen-experienced T-cell fractions. Of the CD44^{+} cells, PD-1^{+} and LAG-3^{+} expressing cells were gated to identify exhausted T cells.

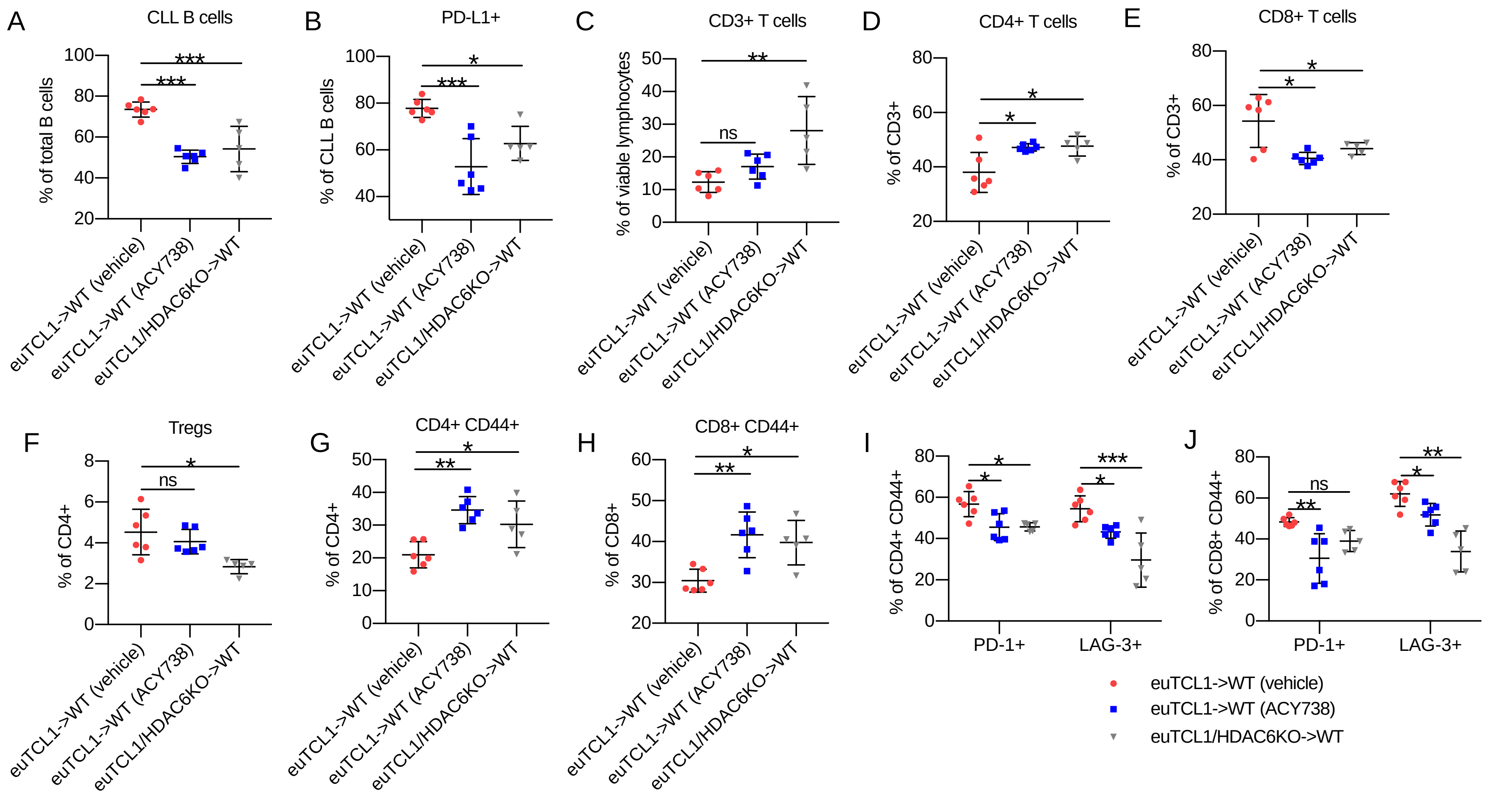

Supplementary Figure 2. CLL immunophenotyping analysis in spleen tissue. (A-J) CLL burden and T-cell subset distribution was determined by flow cytometry analysis in spleen tissue in a cohort of mice sacrificed at Week 7. CLL B cells were gated as $CD3^{-}$ $CD19^{+}$ $B220^{LO}$ IgM^{HI} $CD5^{+}$ cells. Total B cells were gated as $CD3^{-}$ $CD19^{+}$ $B220^{+}$ cells. T cell gating was performed identical to the strategy shown in Supplemental Figure 1. n=5-6 per group. Graphs display mean + SD. *p<0.05, **p<0.005, ***p<0.005. ns: not significant.

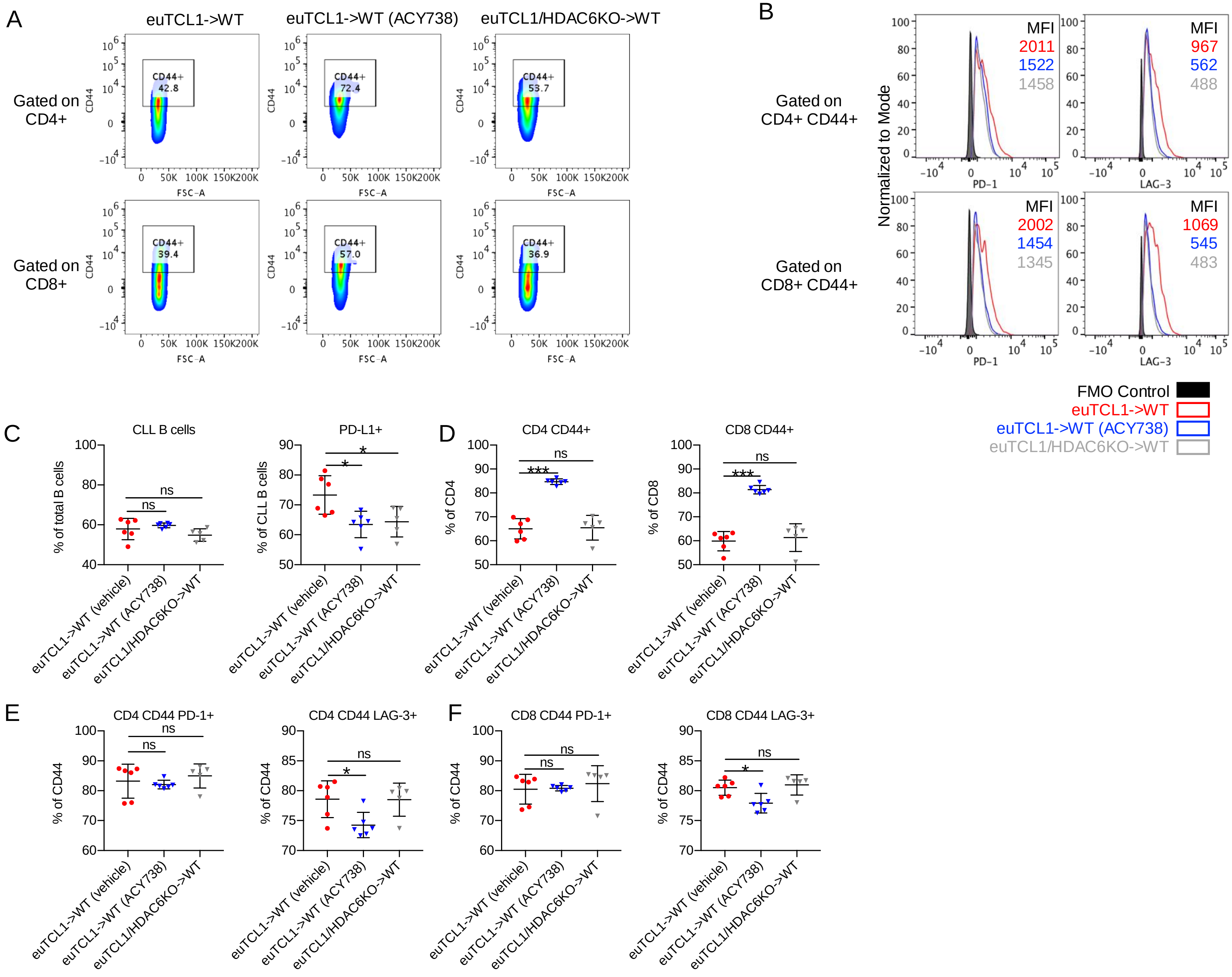

A
euTCL1->WT
euTCL1->WT (ACY738)
euTCL1/HDAC6KO->WT
Gated on CD4+
Gated on CD8+
CD44+ 42.8
CD44+ 72.4
CD44+ 53.7
CD44+ 39.4
CD44+ 57.0
CD44+ 36.9
CD44
FSC-A
B
Gated on CD4+ CD44+
Gated on CD8+ CD44+
Normalized to Mode
MFI 2011 1522 1458
MFI 967 562 488
MFI 2002 1454 1345
MFI 1069 545 483
PD-1
LAG-3
FMO Control
euTCL1->WT
euTCL1->WT (ACY738)
euTCL1/HDAC6KO->WT
C
CLL B cells
PD-L1+
% of total B cells
% of CLL B cells
D
CD4 CD44+
CD8 CD44+
% of CD4
% of CD8
E
CD4 CD44 PD-1+
CD4 CD44 LAG-3+
F
CD8 CD44 PD-1+
CD8 CD44 LAG-3+
% of CD44
euTCL1->WT (vehicle)
euTCL1->WT (ACY738)
euTCL1/HDAC6KO->WT
ns
*
***

Supplementary Figure 3. Representative flow cytometry plots showing (A) Percentage of CD44$^+$ cells in peripheral blood samples from each experimental group and (B) PD-1 and LAG-3 expression on CD44$^+$ cells gated in A. (C-F) Mice with similar tumor burden from the 10-week time point represented in Figure 1B were compared, n=5-6 mice per group. CLL burden all groups range 49-62.8%, mean 57.8%, SD 3.91%. Graphs display mean + SD. *p<0.05, **p<0.005, ***p<0.005. ns: not significant.

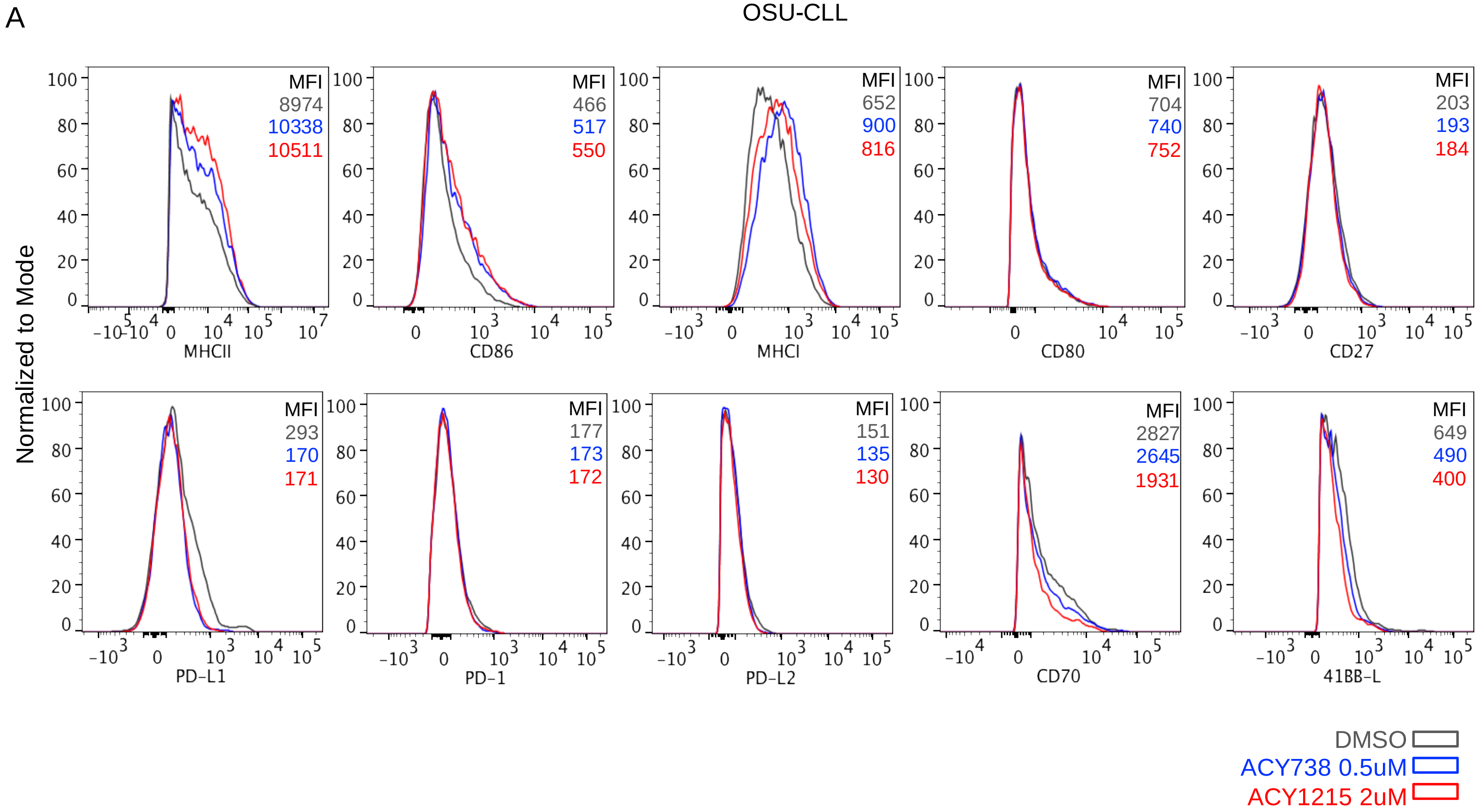

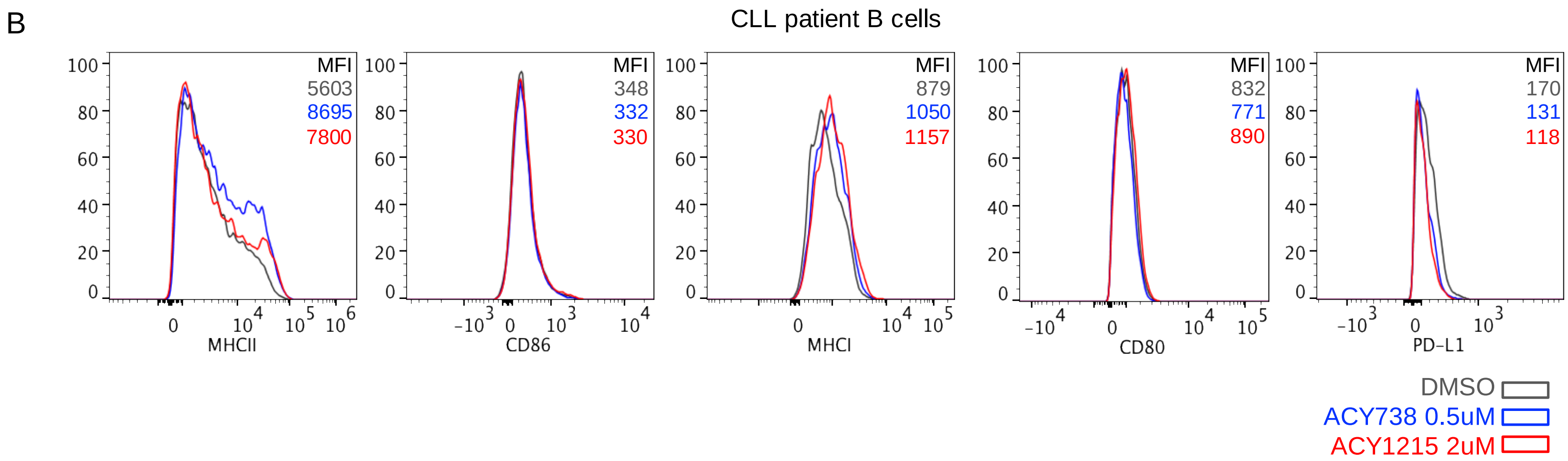

Supplementary Figure 4. Representative histograms showing expression of antigen presentation, co-stimulatory/co-inhibitory molecules on the surface of (A) OSU-CLL cell line and (B) previously frozen primary CLL patient B cells obtained from peripheral blood mononuclear cells. OSU-CLL was gated on singlet viable cells. CLL patient B cells were gated on singlet, viable, $CD19^{+}$ $CD20^{+}$ $CD5^{+}$ cells.

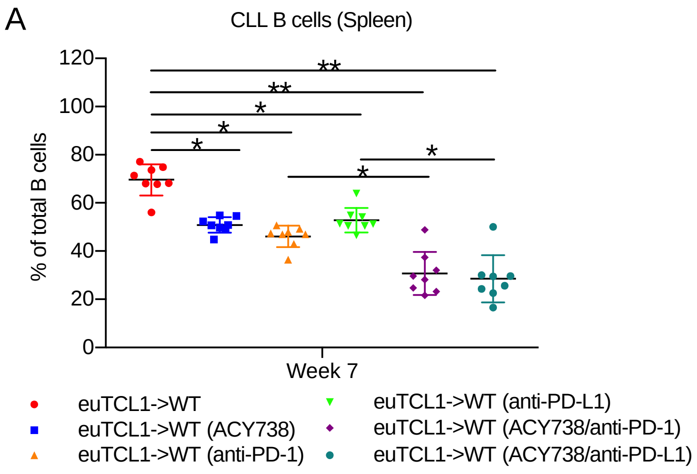

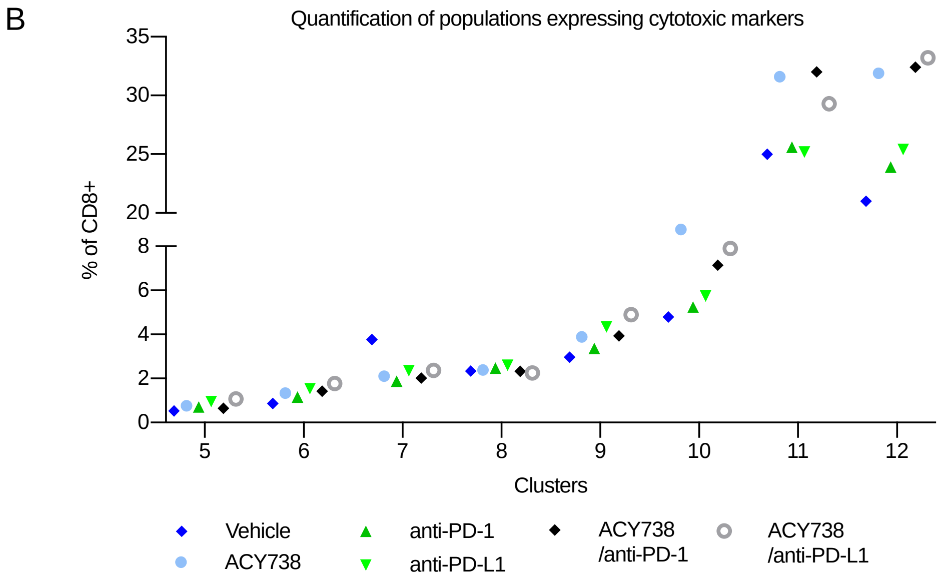

Supplementary Figure 5. Xshift analysis (A) Tumor burden analysis in splenocytes. Graphs display mean + SD. *p<0.05, **p<0.005, ***p<0.005. n=8 mice per group. (B) Quantification of populations expressing cytotoxic markers outlined in Figure 6A.

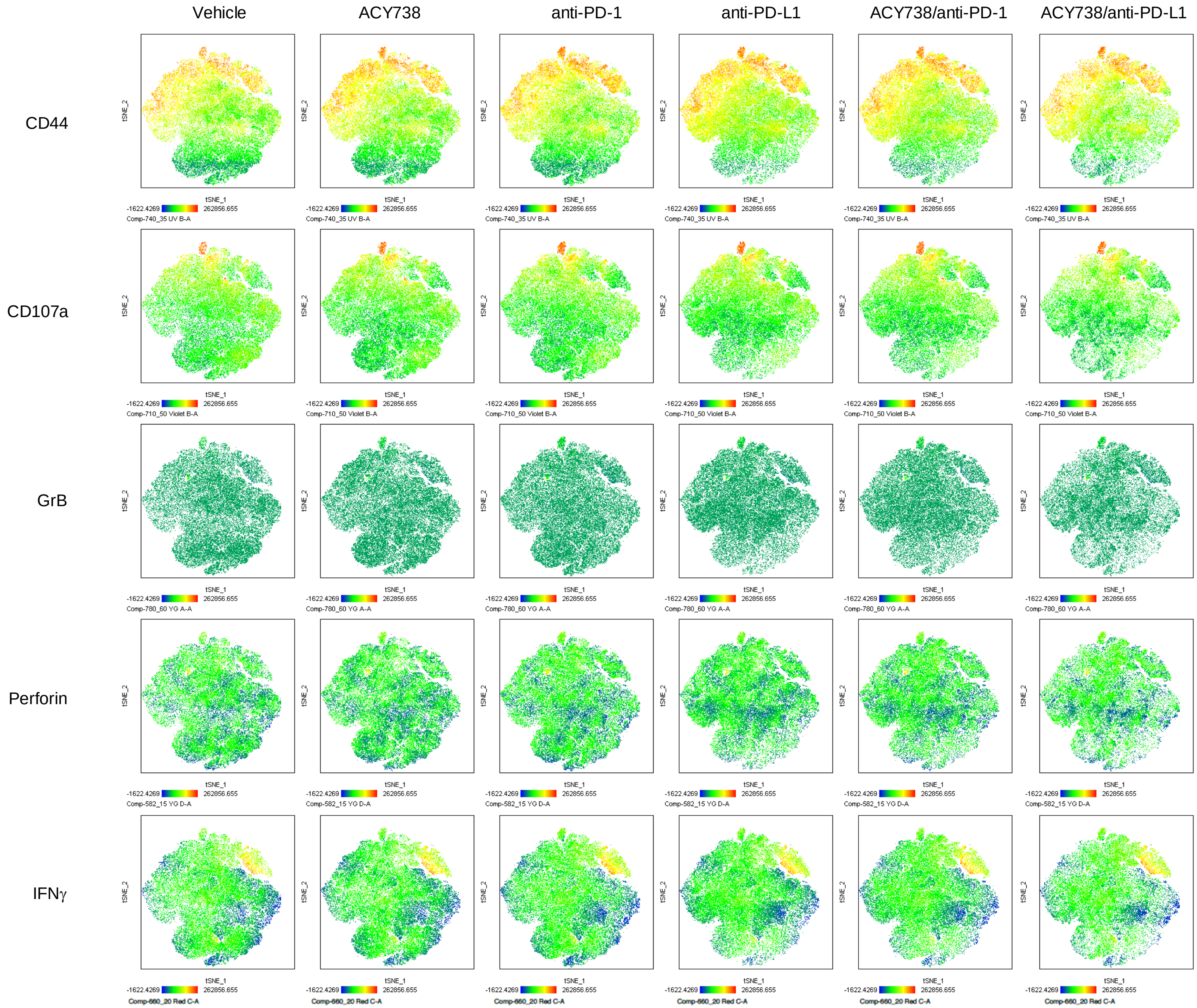
Vehicle
ACY738
anti-PD-1
anti-PD-L1
ACY738/anti-PD-1
ACY738/anti-PD-L1
CD44
CD107a
GrB
Perforin
IFNγ
tSNE_1
tSNE_2
-1622.4269
262856.655
Comp-740_35 UV B-A
Comp-710_50 Violet B-A
Comp-780_60 YG A-A
Comp-582_15 YG D-A
Comp-660_20 Red C-A

Supplementary Figure 6. tSNE plot showing expression of each marker in the $CD8^+$ population. Heat map below each plot shows the level of expression where blue = low and red = high.

Supplementary Table 1. List of flow cytometry and western blot antibodies

ANTIBODIES	SOURCE	IDENTIFIER
Anti-mouse CD19 (1D3) Alexa Fluor 700	BD Biosciences, San Jose, CA	Cat# 557958
Anti-mouse/human CD45R/B220 (RA3-6B2) Alexa Fluor 488	Biolegend, San Diego, CA	Cat# 103225
Anti-mouse IgM (R6-60.2) PE	BD Biosciences, San Jose, CA	Cat# 553409
Anti-mouse CD5 (53-7.3) PerCP	BD Biosciences, San Jose, CA	Cat# 553025
Anti-mouse CD3e (145-2C11) BV786	BD Biosciences, San Jose, CA	Cat# 564379
Anti-mouse CD274/PD-L1 (MIH5) PE-Cyanine7	eBioscience, San Diego, CA	Cat# 25-5982-82
Anti-mouse CD8a (53-6.7) FITC	BD Biosciences, San Jose, CA	Cat# 553031
Anti-mouse/human CD44 (IM7) Alexa Fluor 700	eBioscience, San Diego, CA	Cat# 56-0441-80
Anti-mouse CD25 (PC61) APC	BD Biosciences, San Jose, CA	Cat# 557192
Anti-mouse CD4 (RM4-5) Pacific Blue	BD Biosciences, San Jose, CA	Cat# 558107
Anti-mouse CD223/LAG-3 (C9B7W) PE	Biolegend, San Diego, CA	Cat# 125207
Anti-mouse CD279/PD-1 (29F.1A12) BV605	Biolegend, San Diego, CA	Cat# 135219
Anti-mouse Perforin (S16009B) PE	Biolegend, San Diego, CA	Cat# 154405
Anti-mouse/human Granzyme B (QA16A02) PE-Cyanine7	Biolegend, San Diego, CA	Cat# 372213
Anti-mouse IFN-y (XMG1.2) Alexa Fluor 647	BD Biosciences, San Jose, CA	Cat# 557735
Anti-mouse CD107a/LAMP-1 (1D4B) BV711	Biolegend, San Diego, CA	Cat# 121631
Anti-mouse/rat/human FoxP3 (150D) Alexa Fluor 647	Biolegend, San Diego, CA	Cat# 320013
Anti-human MHCII HLA-DR Alexa Fluor 647	BD Biosciences, San Jose, CA	Cat# 563591
Anti-human CD86 (FUN-1) APC	BD Biosciences, San Jose, CA	Cat# 555660

Anti-human MHCI HLA-ABC APC	BD Biosciences, San Jose, CA	Cat# 562006
Anti-human CD80 Alexa Fluor 647	Biolegend, San Diego, CA	Cat# 305216
Anti-human CD27 (M-T271) APC	BD Biosciences, San Jose, CA	Cat# 558664
Anti-human PD-L1 CD274 (MIH1) APC	BD Biosciences, San Jose, CA	Cat# 563741
Anti-human PD-1 CD279 (MIH4) APC	BD Biosciences, San Jose, CA	Cat# 558694
Anti-human PD-L2 CD273 (MIH18) APC	BD Biosciences, San Jose, CA	Cat# 557926
Anti-human CD70 (Ki-24) PE	BD Biosciences, San Jose, CA	Cat# 561935
Anti-human 41BB-L CD137 (C65-485) PE	BD Biosciences, San Jose, CA	Cat# 559446
Mouse anti-STAT3 (pS727) Alexa Fluor 647	BD Biosciences, San Jose, CA	Cat# 558099
Mouse anti-STAT3 (pY705) PE	BD Biosciences, San Jose, CA	Cat# 612569
Mouse anti-JAK2	Novus Biologicals, Centennial, CO	Cat# NBP2-59451
HSP90 (C45G5) Rabbit mAb	Cell Signaling Technology, Danvers, MA	Cat# 4877S
Monoclonal Anti-GAPDH antibody	Sigma-Aldrich, St. Louis, MO	Cat# G8795
alpha Tubulin (DM1A)	Santa Cruz Biotechnology, Dallas, TX	Cat# sc-32293
aceylated alpha Tubulin (6-11B-1)	Santa Cruz Biotechnology, Dallas, TX	Cat# sc-23950
Rabbit anti-HSP90 (ac Ly294)	Novus Biologicals, Centennial, CO	Cat# NBP1-77944
